# Green Synthesis of Copper Nanoparticles Using Aqueous Extract of *Grevillea robusta*: In Vitro Antimicrobial and In Silico Approach

**DOI:** 10.1002/cbdv.71632

**Published:** 2026-08-26

**Authors:** Gul Naz, Barkat Ullah, Atta Ur Rahman, Wajih Ud Din, Arshad Iqbal, Dur‐E‐Najaf Khan, Samra Irem

**Affiliations:** ^1^ Department of Botany Islamia College University Peshawar Pakistan; ^2^ Department of Botany University of Malakand Chakdara Pakistan; ^3^ Department of Pharmacy Bacha Khan University Charsadda Charsadda Pakistan; ^4^ Department of Biotechnology University of Sargodha Sargodha Pakistan

**Keywords:** bioactivity, *Grevillea robusta*, molecular docking, nanoparticles, phytochemical

## Abstract

Green synthesis of metal nanoparticles using plant‐derived phytochemicals offers an environmentally sustainable approach for developing novel antimicrobial agents. In the present study, copper nanoparticles (CuNPs) were biosynthesized using aqueous extracts of fresh and dried leaves of *Grevillea robusta* with 1 mM copper sulfate under continuous stirring at 80°C. GC–MS profiling identified several bioactive constituents, including palmitic acid and linolenic acid methyl ester, which may contribute to nanoparticle formation and stabilization. Successful synthesis of stable, crystalline, and well‐dispersed CuNPs was confirmed by UV–visible spectroscopy, FTIR, SEM, EDX, and XRD analyses. The biosynthesized CuNPs exhibited broad‐spectrum antimicrobial activity against clinically and agriculturally important bacterial (*Escherichia coli, Xanthomonas axonopodis pv. punicae, Acinetobacter* sp., and *Citrobacter* sp.) and fungal (*Colletotrichum sp*., *Fusarium solani, Fusarium oxysporum, Macrophomina phaseolina*, and *Alternaria alternata*) pathogens, with EC_50_ values ranging from 2.67–7.23 µg/mL and 2.85–12.65 µg/mL, respectively. Furthermore, CuNPs effectively inhibited biofilm and pellicle formation. Molecular docking demonstrated favorable interactions between selected phytocompounds and microbial target proteins, providing mechanistic support for the observed antimicrobial activity. These findings highlight *G. robusta*‐mediated CuNPs as promising eco‐friendly nanomaterials for future antimicrobial applications.

## Graphical abstract

Green‐synthesized copper nanoparticles derived from Grevillea robusta leaf extracts exhibit potent broad‐spectrum antimicrobial, antibiofilm, and antipellicle activities against clinically and agriculturally important pathogens. Molecular docking supports favorable phytocompound–target interactions, highlighting a sustainable plant‐based nanotechnology platform with promising potential for future antimicrobial applications.

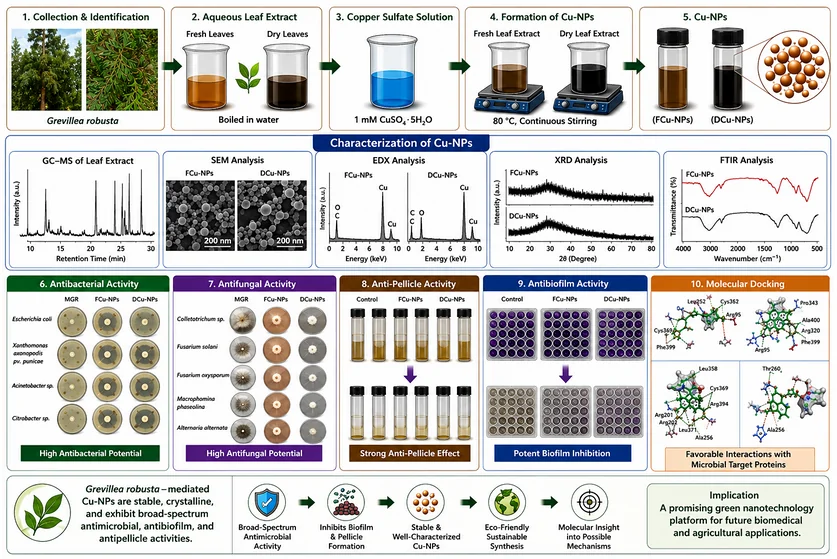

## 1 Introduction

Nanotechnology is an emerging field that encompasses the design, fabrication, and manipulation of materials on a nanoscale, ranging between 1 to 100 nm [1]. During the current period, the preparation of metal and metal oxide nanoparticles has gained full momentum as a notable area among the international scientific community owing to its distinctive physicochemical properties and wide range of applications [2]. These metal and metal oxide nanoparticles are generally used as a key constituent material in a wide range of applications, starting from pharmaceuticals, medicines, agriculture, and textile industries to sensors, electronics, optical fibers, bio‐labeling, production of consumer products, and studies on antimicrobials [3].

A range of methods has been generated for nanoparticle synthesis, which include mainly physical procedures, chemical processes, and biological approaches [4]. The methods include hydrothermal synthesis, precipitation, ultrasonic irradiation, chemical reduction, physical vapor deposition, microemulsion methods, plasma techniques, and sol‐gel techniques [5] of the methods developed, the biological, or green method, has become the method of choice compared to the physical and chemical processes due to the green method's lower cost in terms of energy requirements, cost effectiveness, and environmentally sustainable methods capable of providing stable nanoparticles that are compatible with the biological environment [6]. The green method will facilitate the synthesis of various nanoparticle shapes that include nanospheres, nanorods, nanoporous materials, and nanowires, among others, that are essential for specific applications [7]. The green synthesis of plants, in particular, has been extensively investigated due to the efficiency of plants as biological systems for the production of nanoparticles, thanks to the high diversity of phytochemicals, which act as reducing agents. The green technique utilizing plants has been successfully used for the production of different kinds of metal and metal oxide nanoparticles, including those of gold, silver, silicon, titanium, zinc, copper, palladium, and magnetite [8].

The sudden rise in multidrug‐resistant pathogens has engaged the scientific community for a long period in the quest for new kinds of antimicrobial agents [9, 10]. Metal nanoparticles, among which copper nanoparticles are specifically noted, exhibit broad‐spectrum antimicrobial potential, economic viability, and multi‐target modes for microbial inhibition, including disruption of the membrane, induction of reactive oxygen species, and interference with intracellular processes [11]. Unlike conventional antibiotics, CuNPs show a more multitargeted mode of interaction, hence making resistance less probable [12]. Conventional chemical and physical methods for nanoparticle synthesis do involve toxic reagents and energy‐intensive processes, therefore limiting their environmental and biomedical applications [4]. On the other hand, green synthesis using plant extracts offers a biocompatible, eco‐friendly, and sustainable alternative, using phytochemicals as reducing and stabilizing agents. The preparation techniques not only reduce environmental hazards, but also enhance the stability and bioactivity of the nanoparticles [13].

*Grevillea robusta* is an erect, single‐stemmed tree typically reaching 20–30 m tall and 80 cm in stem diameter. It has a conical crown, dense with dark grey to dark brown bark [14]. Its attractive shape and beautiful foliage make *G. robusta* an ideal ornamental tree for landscaping and roadside plantings as a shade or windbreaks tree, the cut leaves are used in flower arrangements, and young plants are grown as indoor pot plants [15]. The tree has economic potential, which produces an attractively figured, easily worked wood and its branches and twigs are used for firewood and charcoal [16]. The leaves contain several useful chemical compounds, in particular rutin, dyes, phenolic compounds, and gum resin produced from the wood, which have pharmacological applications [17]. *G. robusta*, a bioactive‐rich plant regarding secondary metabolites, is one of those few underexplored candidates for green nanoparticle fabrication. The leaf extract of this plant can act both as a reducing and capping agent for CuNPs and thus might yield nanoparticles with improved antimicrobial activity. Despite being used in medicine, the application of *G. robusta* to the bio‐fabrication of CuNPs and assessment of its antimicrobial activities is not well explored.

This study aimed to investigate the green synthesis of copper nanoparticles using an aqueous leaf extract of *G. robusta*, followed by their physicochemical characterization, in vitro antimicrobial activities against pathogenic microorganisms, and molecular docking analysis in order to investigate possible interactions with bacterial protein targets. The present work combines sustainable nanotechnology, experimental microbiology, and computational modeling to provide a new mechanistically informed strategy for developing environmentally friendly antimicrobial agents, pointing out the potential of plant‐mediated nanoparticles to tackle the emerging challenge of antimicrobial resistance.

## 2 Materials and Methods

### 2.1 Collection of Materials

The chemicals or materials used were provided by the department and included nutrient agar, nutrient broth, potato dextrose agar (PDA), copper sulfate, crystal violet, distilled water, ethanol, and methanol. General laboratory materials and equipment used for nanoparticle preparation, testing, and microorganisms were also used. Moreover, analytical and sterilization equipment, such as a centrifuge, autoclave, oven, incubator, and electric grinder, were also used during the process of experimentation. Before performing any experiment, all materials and equipment were properly cleansed and sanitized to avoid any chance of infection or contamination.

### 2.2 Collection and Identification of Plant

Fresh leaves of *G. robusta* were harvested from Cantonment Board Defence Officers Colony Park, Peshawar, Khyber Pakhtunkhwa, Pakistan. The identity of the plant was authenticated using the Flora of Pakistan (Briq), and further verification was provided by Dr. Naveed Akhtar, Associate Professor of Botany, Islamia College Peshawar, Pakistan. The plant was given a voucher number (GR‐Bot‐12052023) and the sample was deposited in the Herbarium, Department of Botany, Islamia College Peshawar (Figure S1).

### 2.3 Preparation of Plant Extract

In preparing the fresh leaf extract, 100 g of fresh, healthy, and green *G. robusta* leaves were washed with tap water followed by distilled water to remove any dust and debris. The fresh leaves were then smashed using a pestle and mortar, along with 400 mL of distilled water, to make a mixture. In preparing the dry leaf extract, fresh leaves were left to air‐dry under shade at room temperature for 10–15 days until they were completely dried. The dried leaves were then powdered to a fine paste using an electric grinder, with 20 g of which a mixture with 200 mL of distilled water was made. Both extracts were boiled at a temperature of 50°C for 30 min while stirred with a sterile glass stirrer until a solution with a brown color was formed. The solution was then allowed to cool, followed by filtration with Whatman No. 1 filter paper to separate the plant extract, which was then stored for further use [18] (Figure S2).

### 2.4 Phytochemical Profiling Using GC‐MS

GC‐MS analysis was used to establish the chemical content of the crude methanolic leaf extract of *G. robusta* in accordance with the procedures described in [19] at the Medicinal Botanic Centre, PCSIR Labs Complex, Peshawar, Pakistan, utilizing a Shimadzu GC‐MS‐QP2010 Plus instrument. Before this analysis, filtration of the extract was carried out, and the appropriate dilation was made using methanol of analytical grade, which was thereafter poured into a clean GC vial. This was similarly injected splitless, but the temperature of the injector was maintained at 250°C, in addition to a total carrier gas flow of 40.0 mL/min. Initially, the temperature in the column oven was set to 50°C, which was incremented at a rate of 6°C/min to eventually reach a temperature of 230°C. This mass spectrometric analysis was carried out in an electron impact mode, characterized by a complete scan across a range of 40–800 *m/z*, but the temperatures of both the ion source and interface were set to 280°C. This was done after the commencement of data acquisition, which was set to begin at 4.10 min and finally ceased after 29.60 min. This identification of the chemical content occurring in *G. robusta* was achieved after the comparison of their masses and retention time to the standards found in the library.

### 2.5 Preparation of Copper Sulfate Solution

A stock solution of copper sulfate of 1 mM was prepared by accurately weighing 4 mg of copper (II) sulfate pentahydrate, CuSO_4_·5H_2_O, using an analytical balance. The filtered weighed salt was transferred to a 500 mL beaker and followed by the addition of 100 mL of distilled water. The resulting mixture was stirred well with a clean glass rod till the entire amount was dissolved. The solution was later kept at ambient temperature in a clean and duly labeled vessel for subsequent experimental use (Figure S3).

### 2.6 Synthesis of CuNPs

CuNPs were prepared according to the procedure reported by [20] with slight modifications. The pre‐prepared copper sulfate solution with a concentration of 1 mM was poured into a 500 mL beaker and then subjected to heating at a temperature of 80°C using a hot plate with continuous stirring using a magnetic stirrer. The aqueous extract of *G. robusta* leaves was slowly added to the solution while continuing with the stirring process. The result showed a brown to black‐green transformation, ensuring the formation of copper nanoparticles. The mixture that contained the copper nanoparticles was then subjected to centrifugation using a speed of 4000 rpm for the separation of copper nanoparticles. The separated copper nanoparticles were then subjected to washing using distilled water three times to ensure the elimination of impurities. Ethanol in low amounts was then added, and the mixture was subjected to a water bath set at a temperature of 65°C to allow the ethanol to evaporate. The copper nanoparticles were then dried using an oven set to 60°C for a period of 15 min before they were stored for further analysis.

### 2.7 Characterization of CuNPs

The CuNPs were characterized for physicochemical and morphological properties. UV–vis spectroscopy was employed to detect the presence and stability of nanoparticles using the surface plasmon resonance property. FTIR analysis allowed identifying the functional groups on the surface of nanoparticles and the chemical bonding mechanism adopted for the stabilization process. SEM analysis allowed observing the size and shape of the nanoparticles and the surface morphology. Energy Dispersive x‐ray analysis, along with SEM, allowed tracing the elemental composition, hence confirming the presence of copper. The crystalline properties and phase structures of the nanoparticles were observed with the help of x‐ray Diffraction analysis based on the procedure illustrated by [21].

### 2.8 Collection and Preservation of Microorganisms

#### 2.8.1 Bacterial Cultures

Bacterial strains were collected from Khyber Teaching Hospital (KTH), Peshawar, such as *Escherichia coli*, (ATCC‐25922), *Xanthomonas axonopodi* (ATCC‐19312)*, Acinetobacter* sp (ATCC‐31299)*, and Citrobacter* sp (ATCC 51641). To keep the bacterial cultures pure and viable, nutrient broth was used for their preservation. For fresh culturing, bacterial strains were spread on nutrient agar plates, which were then incubated at 37°C for 24 h to develop colonies, which were further observed and kept at 4°C for future use. Fungal strains, including *Colletotrichum* sp, *Fusarium solani, Fusarium oxysporum, Macrophomina phaseolina* and *Alternaria alternata* were provided by the Department of Plant Pathology, The University of Agriculture, Peshawar. The actively growing mycelial parts of each fungal culture were transferred aseptically to a new Potato Dextrose Agar (PDA) plate using an inoculation loop. This was done at temperatures of 25°C and was allowed to grow for 5–7 days. Subsequent to adequate development, the fungal strains were stored

### 2.9 Antimicrobial Assay

#### 2.9.1 Antibacterial Activity

The antibacterial potency of synthesized copper nanoparticles and crude extract of *G. robusta* plant leaves was tested by agar disc diffusion technique, as described by [22] with some modifications. Nutrient agar medium was prepared by dissolving 14 g/500 mL of distilled water, then autoclaved at 121°C, 15 psi for 15 min. About 20 mL of autoclaved Petri dishes were filled with molten agar and allowed to solidify. The bacterial cultures were homogeneously spotted on agar surfaces with cotton swabs that were autoclaved. Stock solution of CuNPs and crude extracts of *G. robusta* were prepared in distilled water, but for nanomaterials synthesized from extracts of dry plant leaves, DMSO (dissolved in water) was used. Concentrations of test media of 3, 6, 9, and 12 µg/ml were prepared. Filter paper discs of 6 mm were sterilized on agar surfaces and soaked with test sample preparations. Ciprofloxacin antibiotic act as a standard positive control. After 24 h of incubation at 37°C, measurements of inhibition zones were recorded in millimeters from three different points.

#### 2.9.2 Antifungal Properties

Antifungal activity was evaluated through a scaled‐down version of the disc diffusion technique proposed by [23]. PDA agar was obtained through the dissolution of 19.5 g in 500 mL distilled water and was auto sterilized using an autoclave. After solidification, the agar was inoculated with the newly obtained fungal samples. Sterilized 6 mm discs of filter paper soaked with newly prepared leaf nanoparticles, dry leaf nanoparticles, and crude extracts of concentrations 3, 6, 9, and 12 µg/mL, respectively, were placed on the agar surface. Fluconazole was used as a positive control. Incubation was performed at 25°C for one week, and the zone sizes were expressed in mm.

### 2.10 Anti‐Pellicle

The antipellicle activity was assessed based on the method described by [24] with some modifications. The test tubes were filled with 3 mL of sterilized nutrient broth, followed by the addition of several drops of 24 h‐old bacterial cultures. The test concentrations of 15, 30, and 45 µg/mL CuNPs and crude extract were used. Ciprofloxacin antibiotic was used as the positive‐control agent. The test tubes were incubated for seven days at 37°C. The development of pellicles was observed. If the pellicle formed was ++++, ++, +, or –, it showed low, medium, strong, or complete antipellicle activity, respectively. If the pellicle leaked out, it showed extremely strong antipellicle activity.

### 2.11 Antibiofilm Activity

Antibiofilm activity was determined through the use of a microtiter plate test based on a modification of [25] a sterilized 96‐well plate made of polystyrene was used, and each well contained 180 µL of nutrient broth and 10 µL of a fresh bacterial suspension. Solutions of CuNPs and crude methanolic extracts were prepared at concentrations of 3, 6, 9, and 12 µg/mL, and 10 µL aliquots were placed in the designated wells. Ciprofloxacin antibiotic served as the positive control, while the negative control entailed the use of wells filled only with broth and bacteria. The plates were incubated for 24 h at 37°C. After the incubation process, the wells were washed using distilled water, and the surface‐dried samples were stained for 10–20 min using a solution of 0.1% crystal violet. The excess stain was drained, and the wells were washed using diluted ethanol. Biofilm formation was quantified using the optical density measured through the use of a TC‐96 ELISA microplate reader at a range of wavelengths from 570 to 630 nm.

### 2.12 Molecular Docking

#### 2.12.1 Docking and Simulation

##### 2.12.1.1 Target Protein Receptor

**FIGURE 1:**
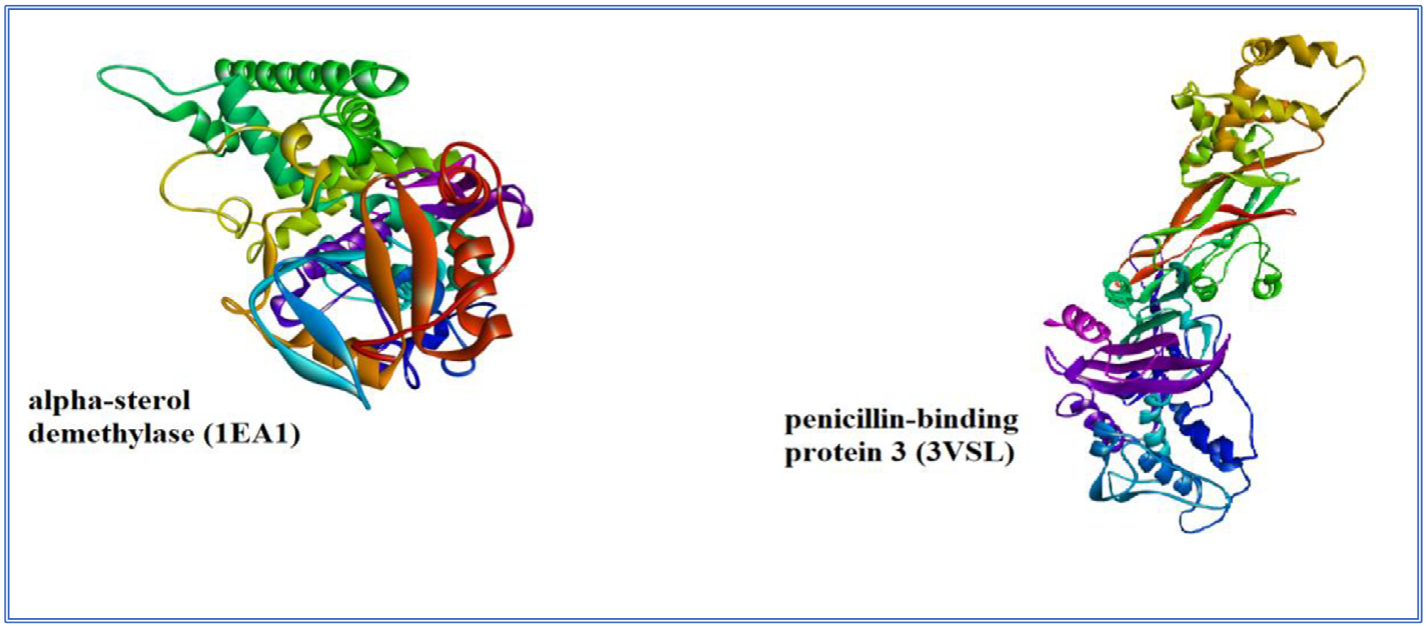
X‐ray crystallographic structure of alpha‐sterol demethylase and penicillin binding protein 3.

Molecular docking studies were conducted to evaluate the antibacterial and antifungal potential of bio‐fabricated CuNPs from *G. robusta* leaf extract. The selected target proteins included alpha‐sterol demethylase (PDB ID: 1EA1) for antifungal activity and penicillin‐binding protein 3 (PDB ID: 3VSL) for antibacterial activity. The 3‐D structures of these proteins were obtained in PDB format from the RCSB Protein Data Bank (https://www.rcsb.org) for further analysis (Figure 1).

#### 2.12.2 Protein Preparation

The three‐dimensional crystal structures of the target proteins, determined by X‐ray crystallography (PDB codes 1EA1 and 3VSL), were obtained from the Protein Data Bank database. Preparation of proteins was performed using the Molecular Operating Environment software, version 2015.10. First, unwanted water molecules were removed, while the structurally relevant water molecules participating in bonding with residues in the active site of proteins were kept. Hydrogen atoms were added to stabilize molecules, and missing side chains and atoms were repaired. Subsequently, partial atomic charges were assigned to improve the accuracy of docking. Protein structures were then refined by energy minimization using the molecular Mechanics engine of the Molecular Operating Environment software with the MOE force field. The energy‐minimized protein structures were saved in PDB format, according to the procedure followed by [26].

#### 2.12.3 Active Site Identification

Active sites of target proteins were modeled using Site Finder software incorporated within the MOE software package, which was further validated with the CASTp web server at the given link‐(http://cast.engr.uic.edu), as cited by Khan et al. (2022). CASTp results yielded data on the presence of pocket and internal regions on the protein structure and other details with respect to residues involved for each site. The molecular docking method calculated the surface area and volume of the pocket using both Solomon‐Richards and Connolly molecular models. The results for active sites were then chosen for molecular docking analysis to test ligand‐protein interaction [27].

#### 2.12.4 Target and Ligand Optimization

Bioactive phytochemicals reported from *G. robusta* were identified using published literature and retrieved from the PubChem database (http://pubchem.ncbi.nlm.nih.gov/). Ligand preparation was performed using MOE's automated protocol, which included three‐dimensional protonation and energy minimization. The optimized ligand structures were then converted into PDB format to ensure compatibility with docking simulations and minimization. The optimized ligand structures were further translated into the PDB format.

#### 2.12.5 Molecular Docking Analysis

Molecular docking analysis was performed to assess the binding affinity, binding energy, and best conformation of binding for the phytochemicals derived from *G. robusta* and copper nanoparticles with the isolated target proteins. Flexible docking analysis was employed by generating multiple conformation structures for the ligands to enhance the accuracy of prediction [28]. Rescoring techniques 1&2 were used for optimizing the outcomes of the docking analysis. The interactions between the ligands and proteins, such as hydrogen bonding and hydrophobic interactions, were assessed by the visualization software BIOVIA Discovery Studio Visualizer version 21.1.0.20298.

#### 2.12.6 Pharmacokinetic Analysis

The pharmacokinetic parameters of the identified phytochemicals were carried out using the online tool SwissADME. The parameters that this tool evaluates include molecular weight, octanol/water partition coefficient (QPlogPo/w), number of hydrogen bond acceptors and donors, and human oral absorption. A drug likeness analysis based on Lipinski's rule of five criteria was also conducted to evaluate whether these phytochemicals can be pursued as drug candidates [29].

#### 2.12.7 Toxicity Prediction

The toxicity profiles for the chosen ligands were calculated by the web‐based platform ProTox‐II available at http://ox‐new.charite.de/protox, a tool for estimating toxicological endpoints as well as categorizing compounds on the basis of the levels of toxicity they pose.

### 2.13 Statistic Analysis

All experiments were performed in triplicate (*n* = 3), and the results are presented as mean ± standard deviation (SD). Data organization, graphical representation, and descriptive statistical analyses were performed using GraphPad Prism version 8.0.

## 3 Results

In the present study, the leaves of *G. robusta* were investigated for antimicrobial and molecular docking potential. GC‐MS analysis identified phytochemicals in the crude extract. Fresh and dry leaf extracts were used for green synthesis of copper nanoparticles with copper sulfate as a precursor. Nanoparticles were characterized using UV–vis, FTIR, SEM, EDX, and XRD and tested for antimicrobial activities. Molecular docking analyzed phytochemical interactions with microbial target proteins.

### 3.1 GC‐MS Analysis *G. robusta* Leaf Crude Extract

The GC–MS analysis of the *G. robusta* leaf crude extract identified 10 major compounds based on their retention times, peak areas, and relative abundances. The chromatogram (Figure S4) exhibited several peaks corresponding to different compounds, with retention times ranging from 9.623 to 29.376 min. Among the identified constituents, 3‐O‐methyl‐D‐glucose (25.64%) was the most abundant compound, followed by palmitic acid (22.86%) and palmitic acid methyl ester (15.10%). Other notable compounds included decane (9.40%), linolenic acid methyl ester (8.87%), and 1‐octadecyne (6.15%), along with several additional bioactive constituents (Table 1). The presence of fatty acids, including palmitic acid and linolenic acid methyl ester, as well as hydrocarbons and other phytochemicals, highlights the diverse chemical composition of the extract.

**TABLE 1:** GC‐MS analysis of crude methonolic extract of *G. robusta*.

ID#	Compounds	R. Time	Area	Conc. (%)
1	Decane	9.623	103969	9.40
2	Tridecane	12.016	62449	5.64
3	3‐O‐Methyl‐d‐glucose	22.134	283664	25.64
4	1‐Octadecyne	25.098	68048	6.15
5	3,7,11,15‐Tetramethyl‐2‐hexadecen‐1‐o1	25.524	13201	1.19
6	9‐Eicosyne	25.836	20676	1.87
7	Palmitic acid, methyl ester	26.555	167042	15.10
8	Palmitic acid	27.223	253006	22.86
9	9,12‐Octadecadienoic acid, methyl ester	29.272	36372	3.29
10	Linolenic acid, methyl ester	29.376	98096	8.87

### 3.2 UV–Visible Spectroscopy

**FIGURE 2:**
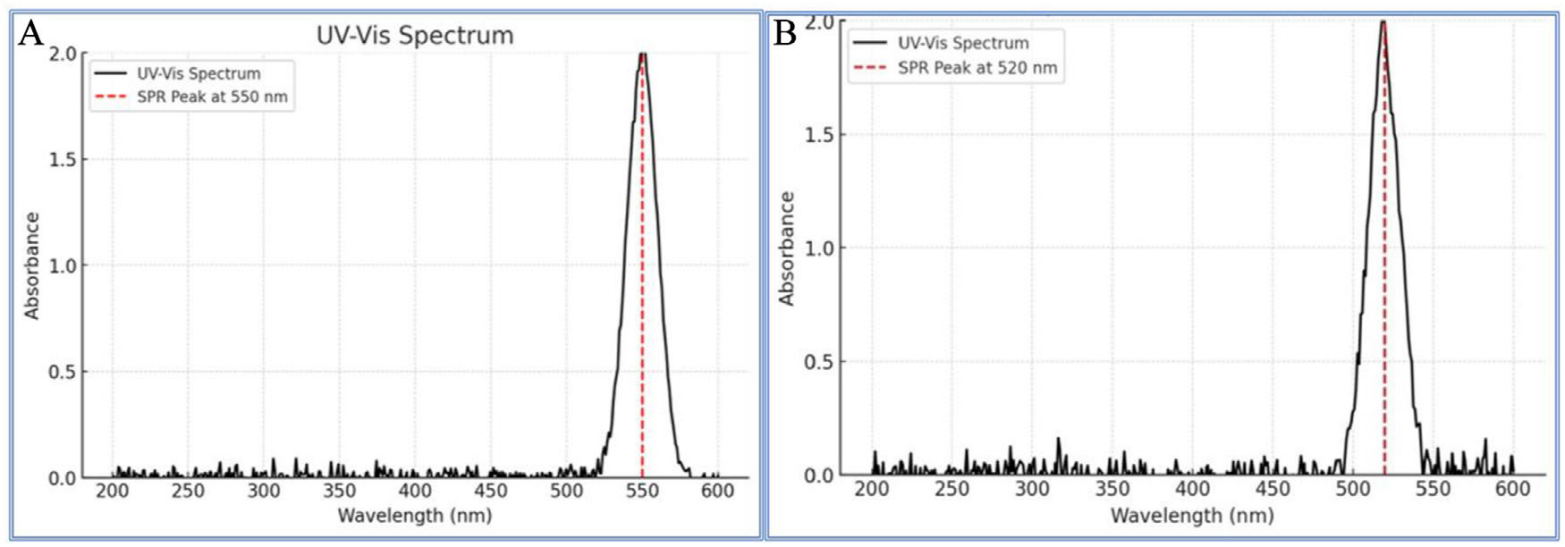
(a‐b) UV–Visible absorption spectra of copper nanoparticles (Cu─NPs) synthesized using fresh and dry leaf extracts of G. robusta.

UV–vis spectroscopy analysis was performed to confirm the synthesis and surface plasmon resonance characteristics of bio‐fabricated CuNPs using UV–Visible Spectrometer at a wavelength of 200–600 nm. The Cu‐NPs synthesized from fresh leaf extract of *G. robusta* showed surface plasmon resonance peak at 550 nm, and CuNPs (dry leaf sample) showed SPR peak at 520 nm (Figure 2).

### 3.3 Formation of Cu─NPs From Fresh and Dry Leaves

**FIGURE 3:**
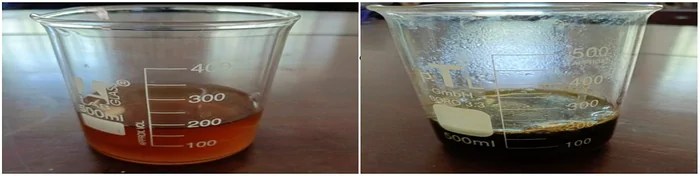
Synthesis and formation of copper nanoparticles (Cu‐NPs) using fresh and dry leaf extracts of G. robusta.

CuNPs were successfully synthesized when aqueous extracts of fresh and dry leaf of *G. robusta* were mixed in 1 mM CuSO4 solution through a bioreduction method. The synthesis process was visually confirmed by observing a color change, where the initial brown color of extract of *G. robusta* gradually changed into blackish‐green by adding 60 mL of plant extract. The color shift showed the bioreduction of copper sulfate (CuSO4) with the help of phytochemicals present in the extract, which leads to the formation of CuNPs. The dark green color suggested the formation of a higher quantity of nanoparticles (Figure 3).

### 3.4 Characterization of Cu‐NPs

#### 3.4.1 SEM Analysis of Fresh and Dry Leaf‐Synthesized Cu─NPs

**FIGURE 4:**
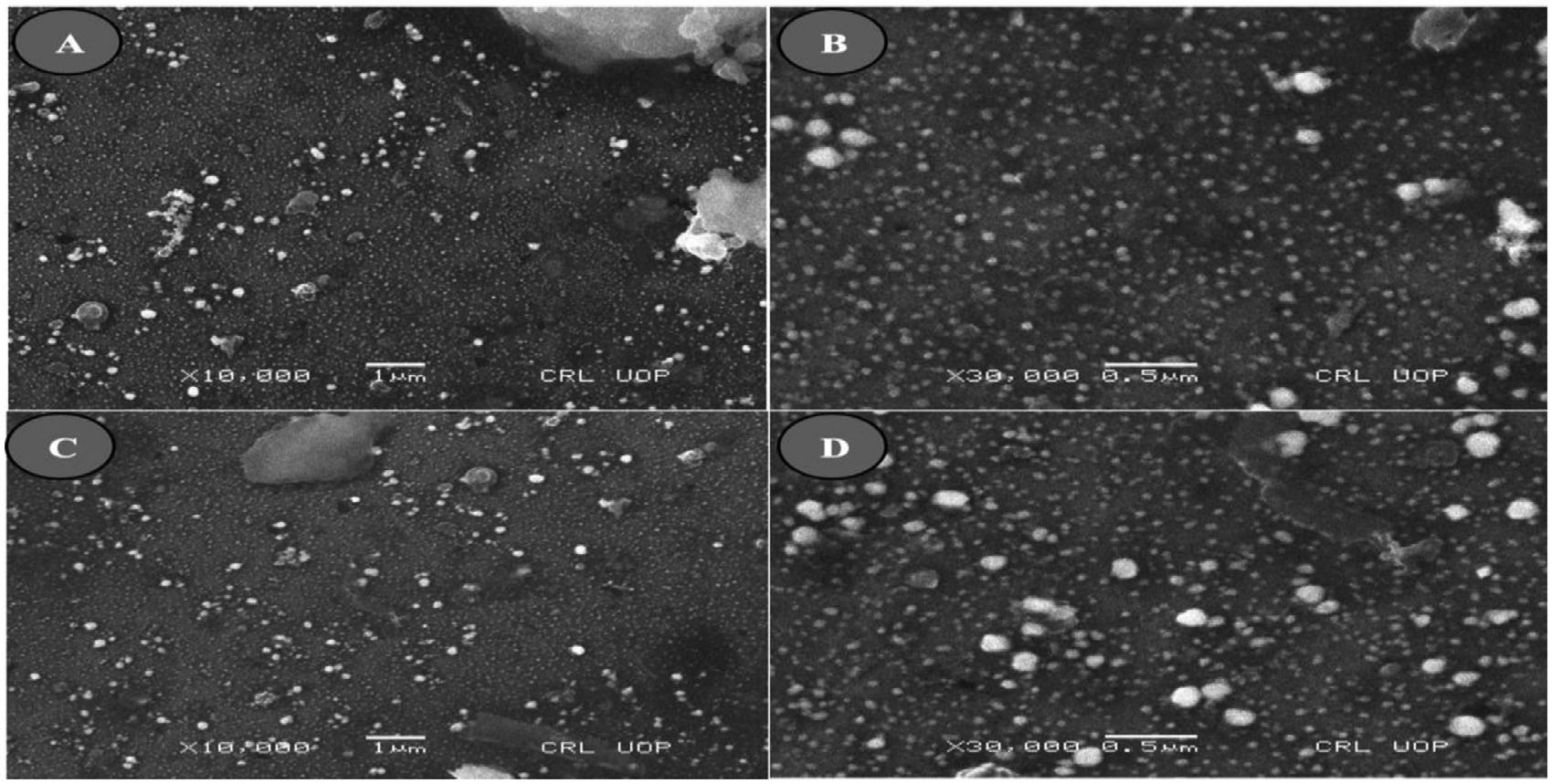
Scanning electron microscopy (SEM) images of biosynthesized Cu─NPs from *G. robusta* leaf extracts at different magnifications: (A) Fresh leaf extract Cu─NPs at ×10 000, (B) Fresh leaf extract Cu─NPs at ×30 000, (C) Dry leaf extract Cu─NPs at ×10,000, and (D) Dry leaf extract Cu─NPs at ×30 000.

Scanning Electron Microscopy (SEM) was employed to investigate the morphology and surface characteristics of Cu─NPs synthesized using fresh and dry leaf extracts of *G. robusta* (Figure 4). SEM micrographs were recorded at magnifications of ×10,000 and ×30,000 corresponding to scale bars of 1 and 0.5 µm, respectively. The Cu──NPs synthesized using fresh leaf extract (Figure 4A,B) exhibited predominantly spherical morphology with relatively uniform distribution and minimal aggregation. At higher magnification, the particles appeared well dispersed, indicating effective stabilization by phytochemicals present in the fresh leaf extract. Similarly, Cu─NPs synthesized using dry leaf extract (Figure 4C,D) displayed predominantly spherical morphology and nanoscale dimensions; however, a relatively greater degree of particle clustering and agglomeration was observed compared with the fresh leaf‐derived nanoparticles.

#### 3.4.2 Energy Dispersive X‐Ray (EDX) Analysis of Cu─NPs Fresh and Dry Leaves

**FIGURE 5:**
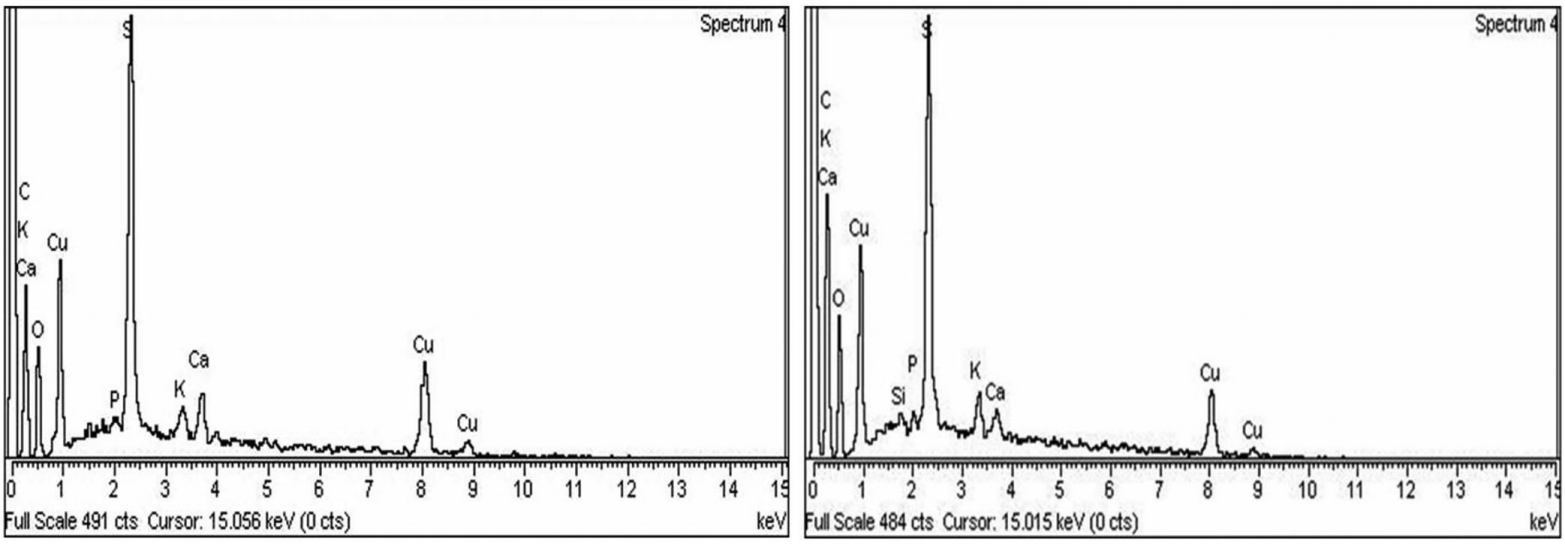
EDX spectra of copper nanoparticles (Cu‐NPs) synthesized using fresh and dry leaf extracts of *G. robusta*.

EDX analysis was performed to determine the elemental composition of Cu‐NPs synthesized using fresh and dry leaf extracts of G. robusta. In the fresh leaf‐mediated Cu‐NPs, carbon (45.17%) and oxygen (19.90%) were the predominant elements, followed by copper (19.22%), sulfur (11.90%), calcium (2.21%), potassium (1.06%), and phosphorus (0.55%). In the dry leaf‐mediated Cu‐NPs, carbon (50.71%) and oxygen (23.16%) were the most abundant elements, followed by copper (11.91%), sulfur (10.97%), potassium (1.40%), calcium (1.01%), phosphorus (0.52%), and a trace amount of silicon (0.30%) respectively, (Table 2, Figure 5).

**TABLE 2:** Elemental composition of Cu‐NPs synthesized from fresh and dry leaf extracts of G. robusta as determined by EDX analysis.

Element	Fresh Leaves		Dry Leaves	
	Weight (%)	Atomic (%)	Weight (%)	Atomic (%)
C	45.17	65.09	50.71	67.14
O	19.9	21.53	23.16	23.02
Si	—	—	0.3	0.17
P	0.55	0.31	0.52	0.27
S	11.9	6.42	10.97	5.44
K	1.06	0.47	1.4	0.57
Ca	2.21	0.95	1.01	0.4
Cu	19.22	5.23	11.91	2.98

#### 3.4.3 X‐Ray Diffraction (XRD) Analysis of Cu‐NPs

**FIGURE 6:**
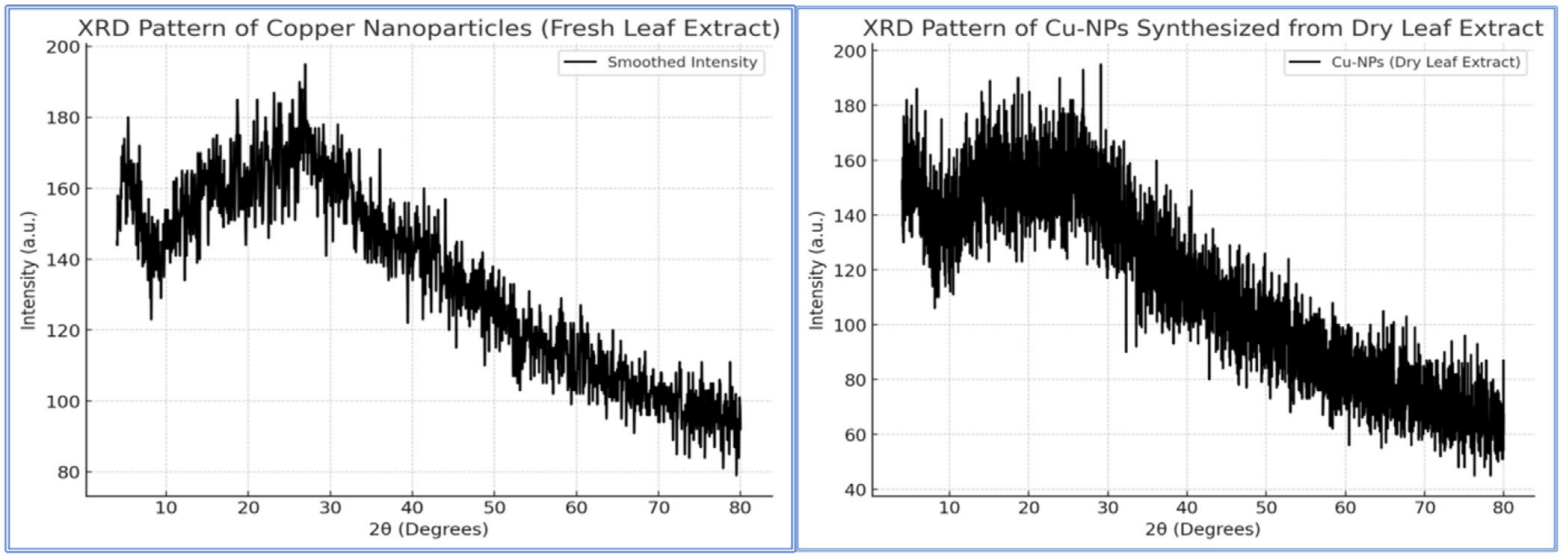
X‐ray diffraction (XRD) patterns of biosynthesized copper nanoparticles (Cu‐NPs) mediated by G. robusta leaf extracts: (A) Fresh leaf extract‐mediated Cu‐NPs and (B) Dry leaf extract‐mediated Cu‐NPs, showing the crystalline nature and phase structure of the synthesized nanoparticles.

X‐ray diffraction (XRD) analysis was performed to determine the crystalline nature of Cu‐NPs synthesized using fresh and dry leaf extracts of *G. robusta* (Figure 6A,B). The XRD pattern displayed different peaks at various 2Ɵ values, confirming the formation of copper nanoparticles. These peaks match to the face‐centered cubic (FCC) structure of copper. The broadness of the peaks shows that the particles are in the nanoscale range. No extra peaks were observed, indicating that the particles are pure without any unwanted impurities.

#### 3.4.4 Fourier Transform Infrared Spectroscopy (FTIR) Analysis of Fresh and Dry Leaf‐Derived Cu‐NPs

**FIGURE 7:**
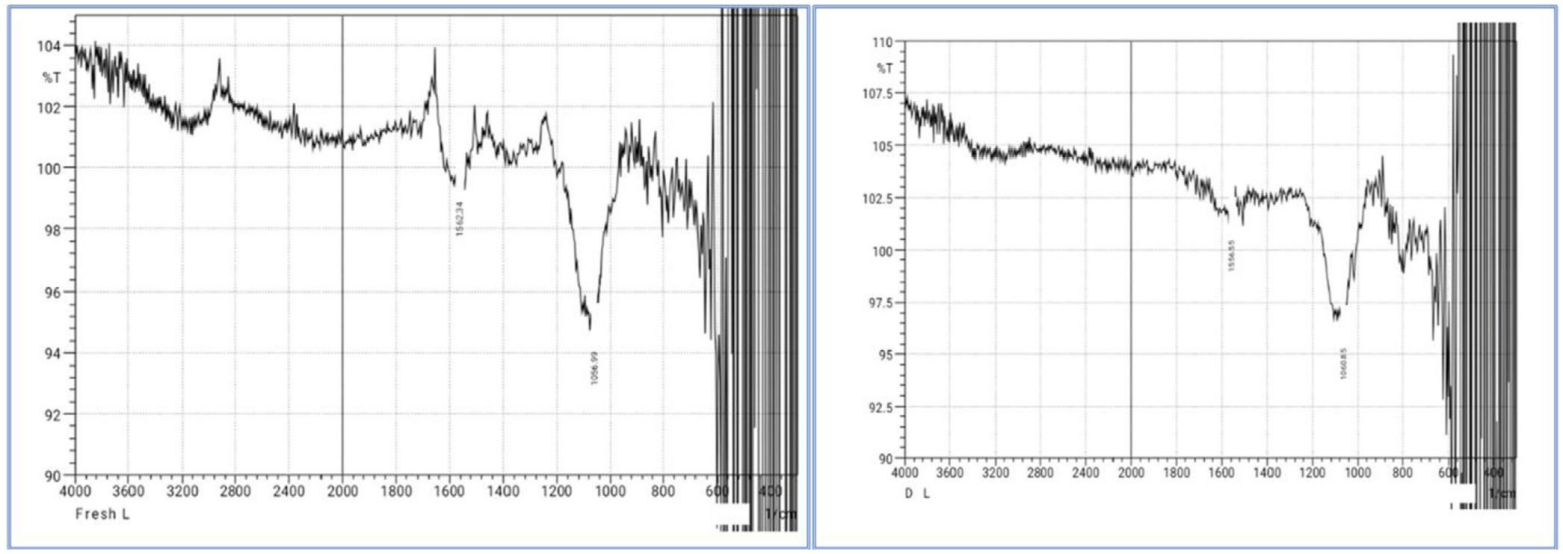
FT‐IR spectra of Cu‐NPs synthesized using fresh and dry leaf extracts of G. robusta. (A) Fresh leaf‐mediated Cu‐NPs and (B) dry leaf‐mediated Cu‐NPs.

The FTIR spectra of Cu‐NPs synthesized from fresh and dry leaf extracts of *G. robusta* are presented in Figure 7, respectively. The band of 1562.34 cm^−1^ was observed for fresh leaf‐derived Cu‐NPs and 1056.99 cm^−1^ was observed for dry leaf‐derived Cu‐NPs and cupric oxide, respectively. The bands at 1562.34 cm^−^^1^ (fresh) and 1556.55 cm^−^^1^ (dry) were assigned to aromatic C═C stretching and/or amide functional groups, while the bands at 1056.99 cm^−^^1^ (fresh) and 1060.85 cm^−^^1^ (dry) corresponded to the stretching vibration of C─O and C─N, respectively.

### 3.5 Antimicrobial Activity

#### 3.5.1 Antibacterial Potential of MGR, FCu‐NPs, and DCu‐NPs

**FIGURE 8:**
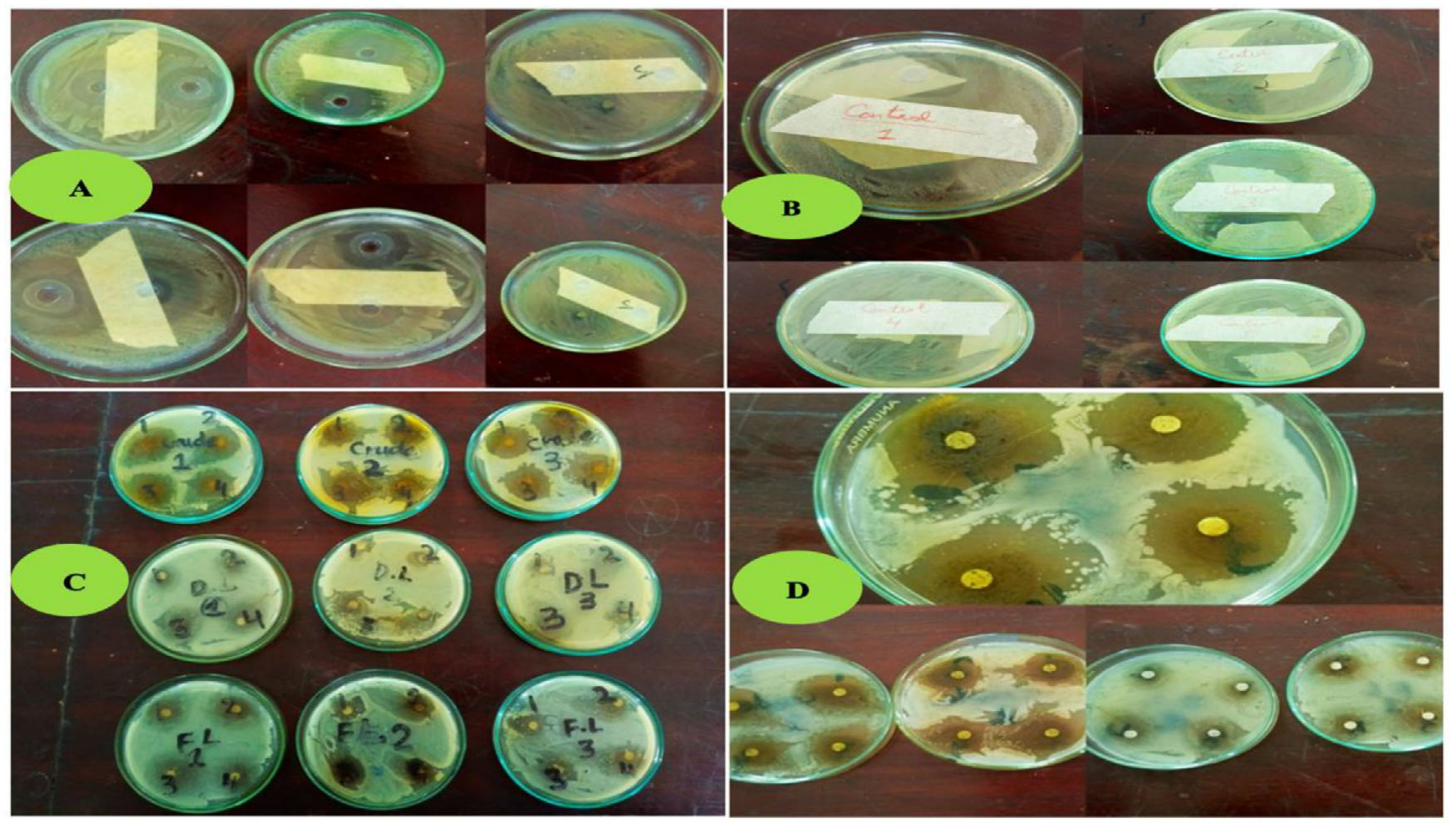
Antibacterial activity of the positive control (Ciprofloxacin) (A–B) and crude extract/Cu‐NPs synthesized from fresh and dry leaf extracts of *G. robusta* (C–D) against 1. *E. coli*, 2. *Acinetobacter* sp., and 3. *Citrobacter* sp. at concentrations of 3, 6, 9, and 12 µg/mL.

The antimicrobial potential of methanolic crude extract (MGR) and copper nanoparticles (Cu‐NPs) synthesized from fresh (FCu‐NPs) and dry (DCu‐NPs) leaf aqueous extracts of G. robusta was evaluated against E. coli, Acinetobacter sp., and Citrobacter sp. using the disc diffusion method, and the half‐maximal effective concentration (EC_50_) was calculated to assess efficacy relative to standard antibiotics mentioned in (Table 3, Figure 8). MGR exhibited moderate antibacterial activity, with inhibition zones against E. coli ranging from 33.67 ± 0.47 mm to 36.67 ± 0.47 mm (EC_50_ = 9.5414), lower than ciprofloxacin (36.67 ± 0.47 mm, EC50 = 5.623), whereas it showed higher effectiveness against Acinetobacter sp. (zones: 31.33–40.67 mm, EC_50_ = 5.8766) and Citrobacter sp. (zones: 30.33–40.00 mm, EC_50_ = 2.6702) compared to the control. FCu‐NPs displayed variable antibacterial activity, with inhibition zones for E. coli, Acinetobacter sp., and Citrobacter sp. ranging from 24.33 ± 0.94 mm to 37.67 ± 0.47 mm, with higher EC_50_ values (7.2358–13.3494), indicating lower efficacy relative to MGR and control for some strains. DCu‐NPs showed reduced antibacterial activity overall, with inhibition zones of 22.33–34.67 mm and EC_50_ values of 6.006–7.2357, being comparatively effective against Acinetobacter sp. and Citrobacter sp.

**TABLE 3:** Antibacterial potential of MGR, FCu─NPs, and DCu─NPs against various bacterial strains.

S. No	Bacterial Strains	Concentration	Control	MGR	FCu‐NPs	DCu‐NPs
1	*Escherichia coli*	3 µg/mL	36.67 ± 0.47	33.67 ± 0.47	27.33 ± 0.47	24.67 ± 0.94
		6 µg/mL	36.67 ± 0.47	36.67 ± 0.47	24.33 ± 0.94	22.67 ± 0.47
		9 µg/mL	36.67 ± 0.47	33.67 ± 0.94	37.67 ± 0.47	34.67 ± 0.47
		12 µg/mL	36.67 ± 0.47	35.33 ± 0.47	27.33 ± 0.94	27.00 ± 0.82
		EC_50_	5.623	9.5414	7.2358	7.2357
2	*Acenetobacter* sp.	3 µg/mL	35.33 ± 0.47	31.33 ± 0.94	25.67 ± 0.47	22.33 ± 0.47
		6 µg/mL	35.33 ± 0.47	35.67 ± 0.47	27.33 ± 0.94	25.00 ± 0.82
		9/mL	35.33 ± 0.47	40.67 ± 0.94	24.67 ± 0.94	35.00 ± 0.82
		12 µg/mL	35.33 ± 0.47	36.33 ± 1.25	36.33 ± 0.47	27.67 ± 0.94
		EC_50_	6.4071	5.8766	12.9772	6.1942
3	*Citrobacter* sp.	3 µg /mL	35.67 ± 0.47	30.33 ± 0.47	28.33 ± 0.47	20.33 ± 0.47
		6 µg/mL	35.67 ± 0.47	40.00 ± 0.00	27.33 ± 0.94	27.33 ± 0.47
		9 µg/mL	35.67 ± 0.47	34.00 ± 0.82	26.67 ± 0.94	35.00 ± 0.82
		12 µg/mL	35.67 ± 0.47	34.33 ± 0.47	32.33 ± 0.47	34.00 ± 0.82
		EC_50_	7.7958	2.6702	13.3494	6.006

All values are expressed as Mean ± S.D and percent inhibition of zone of inhibition in millimeter (mm).

#### 3.5.2 Antifungal Potential of MGR, FCu‐NPs, and DCu‐NPs

**FIGURE 9:**
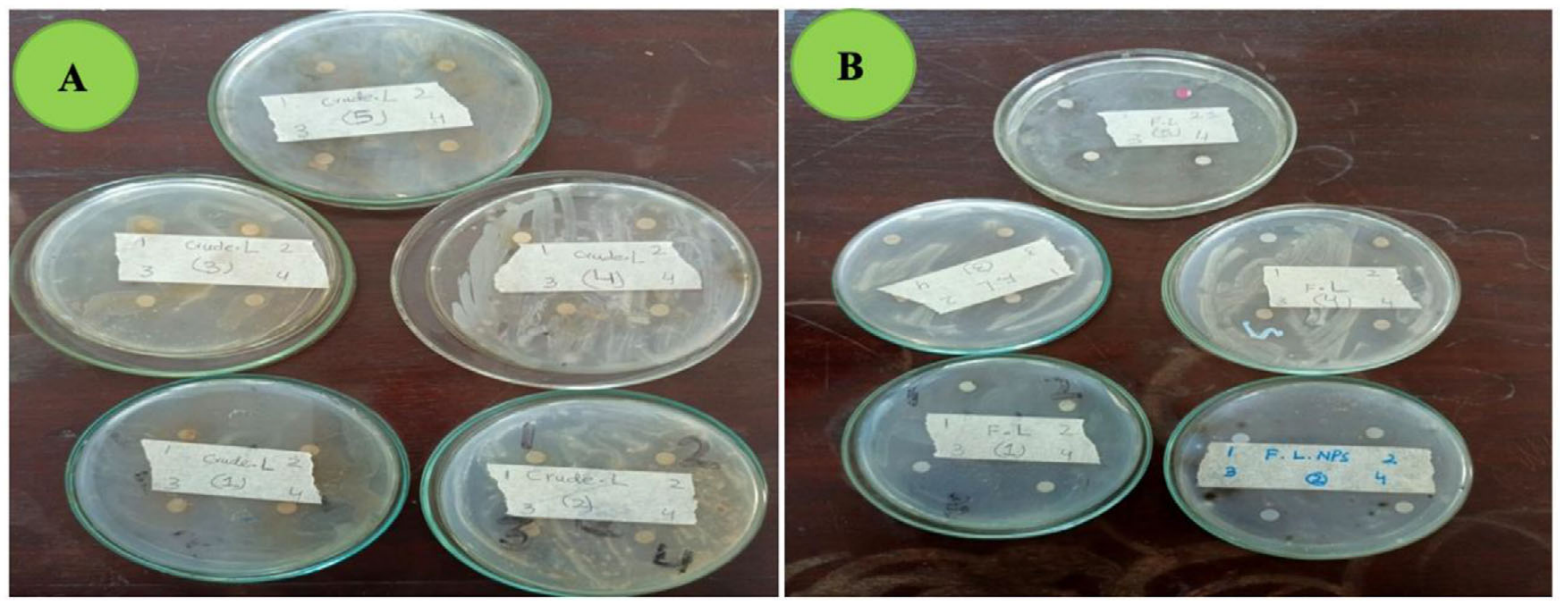
Antifungal activity of the positive controls, Fluconazole and Miconazole (A–B), and methanolic crude extract (MGR) and Cu‐NPs synthesized from fresh and dry leaf extracts of G. robusta (C–D) against 1. Colletotrichum sp., 2. F. solani, 3. F. oxysporum, 4. M. phaseolina, and 5. A. alternata at concentrations of 3, 6, 9, and 12 µg/mL.

The antifungal activity of methanolic crude extract (MGR) and copper nanoparticles synthesized from fresh (FCu‐NPs) and dry (DCu‐NPs) leaf extracts of G. robusta was evaluated against Colletotrichum sp., F. solani, F. oxysporum, M. phaseolina, and A. alternata at concentrations of 3‐12 µg/mL, as mentioned in Table 4, Figure 9. MGR exhibited moderate to high antifungal activity, with notable efficacy against M. phaseolina (zones: 19–34.33 mm, EC_50_ = 2.8506 µg/mL) and substantial activity against F. solani (24.33–27 mm, EC_50_ = 6.0685 µg/mL) and A. alternata (17–26.66 mm, EC_50_ = 6.3239 µg/mL). FCu‐NPs displayed variable inhibition, showing high activity against F. solani (26.33‐36 mm, EC_50_ = 6.0683 µg/mL) and M. phaseolina (28.33‐33.3 mm, EC_50_ = 28.2990 µg/mL), but lower efficacy against F. oxysporum (12–13.66 mm, EC_50_ = 6.0683 µg/mL). DCu‐NPs demonstrated superior antifungal potential overall, with higher inhibition zones and lower EC_50_ values against most strains, including A. alternata (25–30.33 mm, EC_50_ = 18.107 µg/mL) and Colletotrichum sp. (30.66–32 mm, EC50 = 7.3133 µg/mL).

**TABLE 4:** Antifungal potential of MGR, FCu‐NPs, and DCu‐NPs against various fungal strains.

S. No	Fungal strains	Concentration	Control	MGR	FCu‐NPs	DCu‐NPs
1	*Colletotrichum* sp.	3 µg/mL	28.33 ± 1.24	33 ± 2.44	32 ± 1.63	30.66 ± 0.94
		6 µg/mL	28.33 ± 1.24	25.33 ± 1.24	32.66 ± 1.88	32 ± 1.63
		9/mL	28.33 ± 1.24	28 ± 0.94	37 ± 1.41	24 ± 0.81
		12 µg/mL	28.33 ± 1.24	37 ± 1.41	18.66 ± 0.94	26 ± 0.81
		EC_50_	3.4228	12.9375	12.6574	7.3133
2	*Fusarium solani*	3 µg/mL	11.66 ± 0.47	27 ± 0.81	36 ± 1.63	27.66 ± 0.47
		6 µg/mL	11.66 ± 0.47	26.33 ± 1.24	36 ± 0.81	26.33 ± 1.24
		9 µg /mL	11.66 ± 0.47	24.33 ± 1.24	30.66 ± 0.94	26 ± 0.81
		12 µg/mL	11.66 ± 0.47	26.66 ± 1.24	26.33 ± 0.47	20.66 ± 0.94
		EC_50_	9.7545	6.0685	8.9002	15.5315
3	*Fusarium oxysporum*	3 µg/mL	14.33 ± 0.94	39 ± 1.41	12.66 ± 0.47	28 ± 1.41
		6 µg/mL	14.33 ± 0.94	38 ± 0.81	13.66 ± 1.24	29.33 ± 0.94
		9 µg /mL	14.33 ± 0.94	40 ± 1.63	12.33 ± 0.47	30 ± 1.63
		12 µg/mL	14.33 ± 0.94	36 ± 1.41	12 ± 0.81	33 ± 0.81
		EC_50_	6.9011	13.5123	8.5437	22.7066
4	*Macrophomina phaseolina*	3 µg/mL	20 ± 0.81	34.33 ± 1.69	28.33 ± 1.24	27 ± 0.81
		6 µg/mL	20 ± 0.81	19 ± 1.63	30.33 ± 1.24	26 ± 1.63
		9/mL	20 ± 0.81	24.66 ± 2.49	31 ± 1.41	24 ± 0.81
		12 µg/mL	20 ± 0.81	24 ± 1.63	33.33 ± 1.24	23.66 ± 1.24
		EC_50_	13.5862	2.8506	28.2999	6.7646
5	*Alternaria alternata*	3 µg/mL	18 ± 0.81	17 ± 0.81	24.66 ± 0.47	30.33 ± 1.24
		6 µg/mL	18 ± 0.81	19 ± 2.16	18.33 ± 1.24	26 ± 0.81
		9 µg/mL	18 ± 0.81	28.33 ± 1.24	24 ± 0.81	35.66 ± 1.24
		12 µg/mL	18 ± 0.81	26.66 ± 1.24	21 ± 1.63	23 ± 0.81
		EC_50_	9.0708	6.3239	2.6459	13.0127

All values are expressed as Mean ± S.D and percent inhibition of zone of inhibition in millimeter (mm).

### 3.6 Anti‐Pellicle Activity

Anti‐pellicle activity was determined by studying pellicle layer formation with various bacterial strains (Table 5, Figure S5‐S7). *E. coli* (EC) exhibited moderate anti‐pellicle activity (++), and *Acinetobacter* sp. exhibited strong inhibition (+) for Ciprofloxacin (control). The culture samples included (AB) and *Citrobacter* sp. (CB) at 15 µg/mL. The methanolic extract of *G. robusta* had moderate inhibition (++) against EC and CB at 15 and 30 µg/mL and strong inhibition (++) at 45 µg/mL for all the tested bacterial strains. The fresh leaf‐derived Cu‐NPs exhibited the strongest inhibition (+) at 15, 30, and 45 µg/mL against *E. coli*. Moderate inhibition (++) was noted in AB and CB at 15 and 30 µg/mL, respectively, while strong inhibition (+) was noted in CB at 45 µg/mL. The Cu‐NPs obtained from the dried leaves showed weak inhibition (+++) against EC at 15 and 30 µg/mL and moderate inhibition (++) at 45 µg/mL. Strong inhibition (+) was noticed against AB at all the tested concentrations (15, 30, and 45 µg/mL). Moderate inhibition (++) was observed at 15 µg/mL and strong inhibition (+) was observed at 30 and 45 µg/mL under which complete disruption of the pellicle layer was observed for CB.

**TABLE 5:** Anti‐pellicle Activity of Crude extract and Cu‐NPs synthesized from *G. robust* fresh and dry leaf extract.

Sample Treatment	Concentration	*E. Coli*	*Acenetobacter* sp.	*Citrobacter* sp.
Ciprofloxacin (Control)	15 µg/mL	**++**	**+**	**+**
Crude Extract	15 µg/mL	**++**	**+**	**++**
	30 µg/mL	**++**	**+**	**++**
	45 µg/mL	**+**	**+**	**+**
CuNPs Fresh Leaves	15 µg/mL	**+**	**++**	**++**
	30 µg/mL	**+**	**+**	**++**
	45 µg/mL	**+**	**++**	**+**
CuNPs Dry Leaves	15 µg/mL	**+++**	**+**	**++**
	30 µg/mL	**+++**	**+**	**+**
	45 µg/mL	**++**	**+**	**+**

(+): strong inhibition/complete pellicle disruption; (++): moderate inhibition; (+++): weak inhibition; (−): no inhibition.

#### 3.6.1 Antibiofilm Activity

The antibiofilm activity of the methanolic crude extract and Cu‐NPs synthesized from *G. robusta* leaves was evaluated against *Xanthomonas axonopodis pv. punicae, Acinetobacter* sp., and *Citrobacter* sp. using specific biofilm formation (SBF) values. The untreated control exhibited consistently low SBF values (0.004–0.056), indicating minimal biofilm formation under the experimental conditions. The crude extract showed variable effects, with SBF values generally increasing at higher concentrations, particularly against *X. axonopodis pv. punicae* (0.450–0.568), *Acinetobacter* sp. (0.433–0.770), and *Citrobacter* sp. (0.415–0.873), suggesting limited antibiofilm efficacy. In contrast, Cu‐NPs demonstrated lower SBF values at several concentrations. For *X. axonopodis pv. punicae*, the SBF value decreased from 0.413 at 3 µg/mL to 0.261 at 12 µg/mL. Similarly, *Acinetobacter* sp. showed a marked reduction in SBF, reaching 0.036 at 12 µg/mL, while the lowest SBF value for *Citrobacter* sp. was observed at 9 µg/mL (0.003) (Table 6, Figures S8‐S9).

**TABLE 6:** Antibiofilm activity of crude extract and Cu─NPs synthesized from fresh and dry leaf extract of *G. robusta*.

Treatment	Concentration	*Xanthomonas axonopodia*	SBF Value	*Acinetobacter* sp.	SBF Value	*Citrobacter* sp.	SBF value
		OD		OD		OD	
Cell Free							
Cell Growth							
Control	3 µg/mL	0.004	0.014	0.005	0.008	0.006	0.006
	6 µg/mL	0.007	0.008	0.006	0.006	0.005	0.006
	9 µg/mL	0.146	0.029	0.004	0.006	0.008	0.007
	12 µg/mL	0	0.056	0.006	0.008	0.007	0.009
Crude Extract	3 µg/mL	0.006	0.001	0.006	0.013	0.011	0.006
	6 µg/mL	0.007	0.001	0.111	0.256	0.001	0.004
	9 µg/mL	0.645	0.45	0.413	0.433	0.502	0.415
	12 µg/mL	0.538	0.568	0.437	0.77	0.756	0.873
Cu‐NPs(Fresh Leaf)	3 µg/mL	0.553	0.413	0.366	0.246	0.41	0.243
	6 µg/mL	0.359	0.332	0.249	0.545	0.924	0.599
	9 µg/mL	0.422	0.173	0.191	0.081	0.17	0.003
	12 µg/mL	0.271	0.261	0.429	0.036	0.759	0.413
CuNPs (Dry Leaf)	3 µg/mL	0.553	0.413	0.366	0.246	0.41	0.243
	6 µg/mL	0.359	0.332	0.249	0.545	0.924	0.599
	9 µg/mL	0.422	0.173	0.191	0.081	0.17	0.003
	12 µg/mL	0.271	0.261	0.429	0.036	0.759	0.413

### 3.7 Molecular Docking of Cu‐NPs

**FIGURE 10:**
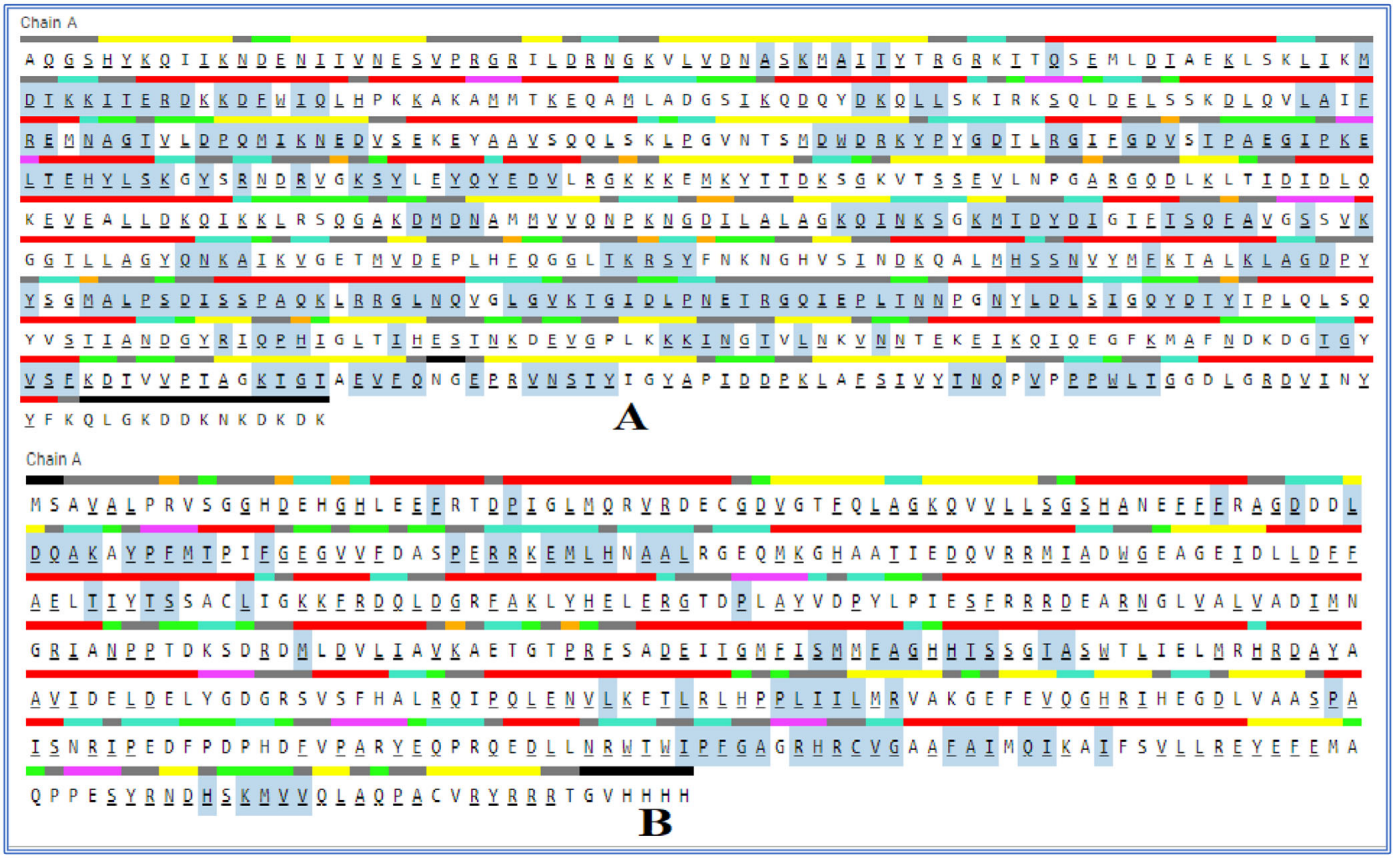
Active site of 1EA1 (Figure A), 3VSL (Figure B) protein found through CASTp server.

**FIGURE 11:**
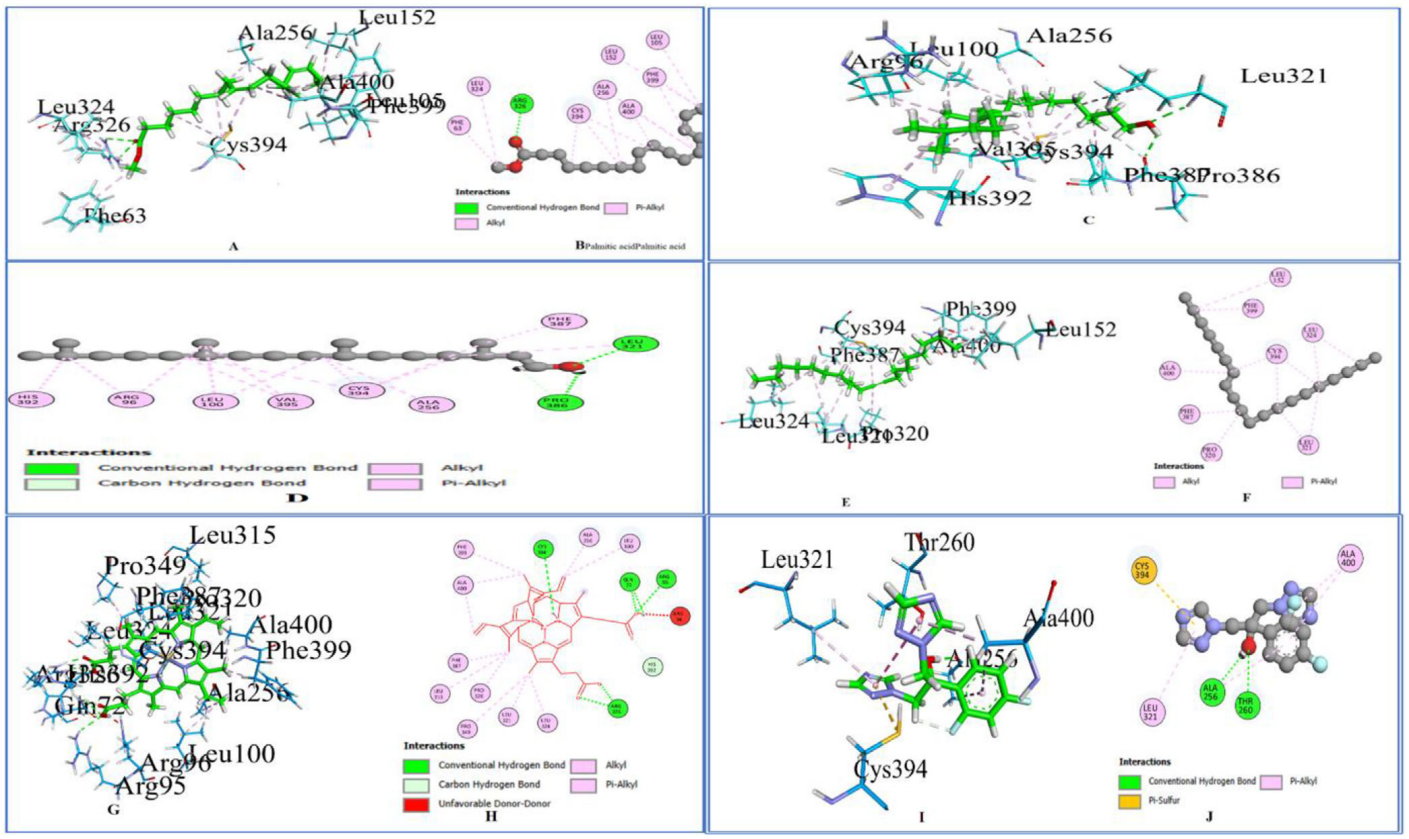
2D & 3D interaction of Linolenic acid, methyl ester (A & B), 3,7,11,15‐Tetramethyl‐2‐hexadecen‐1‐o1 (C & D), 9‐Eicosyne(E & F) inhibitor HEM (G & H) and inhibitor TPF (I & J) with 1EA1.

**FIGURE 12:**
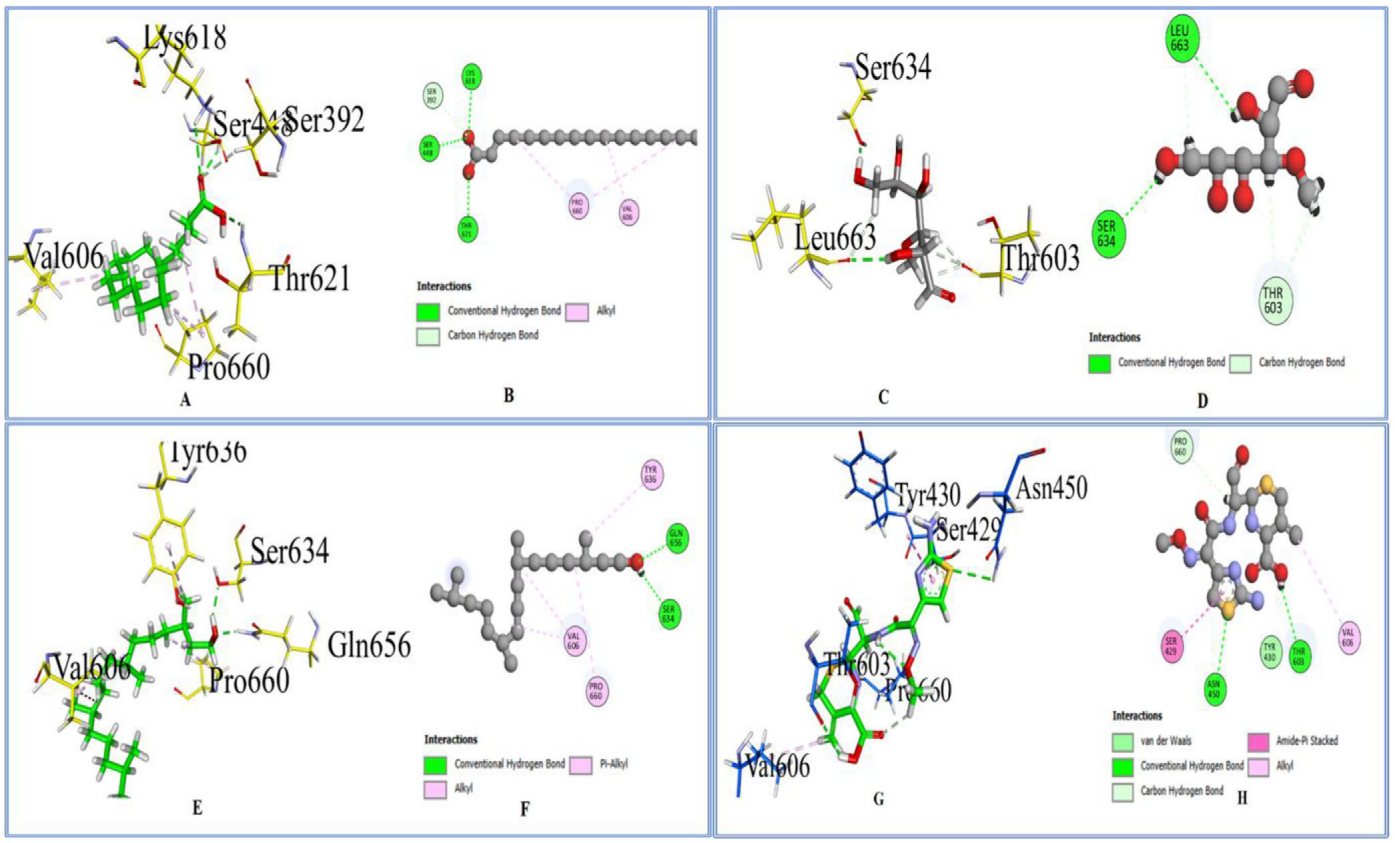
2D & 3D interaction of Palmitic acid (A, B), 3‐O‐Methyl‐d‐glucose (C, D), 3,7,11,15‐Tetramethyl‐2‐hexadecen‐1‐o1(E, F) and inhibitor CEF (G, H) with 3VSL.

Phytochemicals identified from G. robusta were subjected to molecular docking against penicillin‐binding protein (PDB ID: 1EA1), involved in bacterial cell wall biosynthesis, and sterol 14α‐demethylase (PDB ID: 3VSL), a key enzyme in fungal ergosterol biosynthesis. The active binding pockets of both proteins were identified using the CASTp server (Figures 10, 11, 12). Prior to docking analysis, the selected compounds were evaluated for their pharmacokinetic and toxicity profiles. The ADMET analysis revealed that all selected phytochemicals complied with Lipinski's rule of five and exhibited high gastrointestinal absorption, indicating favorable drug‐likeness characteristics (Table 7). Furthermore, toxicity prediction demonstrated acceptable safety profiles, with relatively low predicted hepatotoxicity, neurotoxicity, carcinogenicity, immunotoxicity, mutagenicity, and cytotoxicity for the investigated compounds (Table 8). The docking protocol was validated using the co‐crystallized inhibitors HEM, TPF, and CEF, which exhibited docking scores of −19.58, −10.05, and −14.09 kcal/mol, respectively. Among the tested phytochemicals, linolenic acid methyl ester showed the strongest binding affinity toward 1EA1 (−10.80 kcal/mol), followed by 3,7,11,15‐tetramethyl‐2‐hexadecen‐1‐ol (−10.65 kcal/mol) and 9‐eicosyne (−10.54 kcal/mol). For 3VSL, palmitic acid exhibited the highest binding affinity (−11.27 kcal/mol), followed by 3‐O‐methyl‐D‐glucose (−11.01 kcal/mol) and 3,7,11,15‐tetramethyl‐2‐hexadecen‐1‐ol (−10.11 kcal/mol). A complete summary of the molecular docking scores for all investigated compounds against both target proteins is presented in Table 9. The docked ligands occupied the active‐site regions of both target proteins and formed stabilizing hydrophobic and hydrogen‐bond interactions with surrounding amino acid residues, suggesting their potential to interfere with essential microbial metabolic pathways.

**TABLE 7:** Pharmacokinetic parameters of selected phytochemicals.

ADMET properties	Palmitic acid	Palmitic acid, methyl ester	Linolenic acid, methyl ester	3‐O‐Methyl‐d‐glucose
Molecular weight (g/mol)	256.42 g/mol	270.5 g/mol	292.46 g/mol	256.42 g/mol
TPSA (A°)	37.30 Å^2^	26.30 Å^2^	26.30 Å^2^	37.30 Å^2^
Molar refractivity	80.80	85.12	93.31	80.80
iLOGP	3.85	4.41	5.75	3.58
GI absorption	High	High	High	High
Blood brain barrier (BBB)permeability	Yes	YES	No	Yes
CYP1A2 inhibitor	Yes	Yes	No	Yes
CYP2C19 inhibitor	No	No	No	No
Lipinski	Yes	Yes	Yes	Yes
Log Kp (skin permeation) cm/	−2.77 cm/s	−2.71 cm/s	−7.44 cm/s	−2.77 cm/s

**TABLE 8:** Provides an overview of the anticipated outcomes of the toxicity analysis.

Toxicity Analysis	Hepatotoxicity	Neurotoxicity	Carcinogenicity	Immunotoxicity	Mutagenicity	Cytotoxicity	Radar chart
Palmitic acid	0.52	0.92	0.63	0.99	1.0	0.74	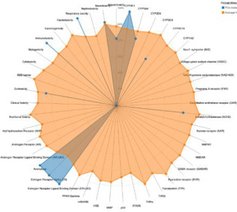
Palmitic acid, methyl ester	0.58	0.80	0.55	0.99	0.98	0.73	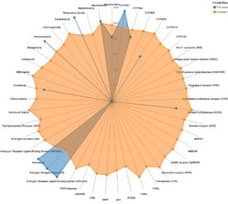
Linolenic acid, methyl ester	0.63	0.83	0.58	0.97	0.83	0.73	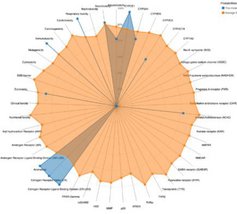
3‐O‐Methyl‐d‐glucose	0.96	0.95	0.83	0.99	0.68	0.84	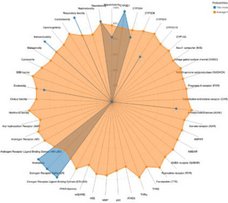

**TABLE 9:** Displaying the docking scores for various compounds.

S.NO	Compounds	Pubhcem ID #	Antibacterial	Antifungal
			(PDB ID: 1EA1)	(PDB ID: 3VSL)
1	Inhibitor (HEM)	—	−19.58	—
2	Inhibitor (TPF)	—	−10.05	—
3	Inhibitor (CEF)	—	—	−14.09
4	Decane	15600	−7.55	−7.05
5	Tridecane	12388	−8.74	−6.51
6	3‐O‐Methyl‐d‐glucose	8973	−8.42	−11.01
7	1‐Octadecyne	69425	−10.26	−7.13
8	3,7,11,15‐Tetramethyl‐2‐hexadecen‐1‐o1	102459	−10.65	−10.11
9	9‐Eicosyne	557019	−10.54	−7.84
10	Palmitic acid, methyl ester	8181	−10.17	−8.39
11	Palmitic acid	985	−9.06	−11.27
12	9,12‐Octadecadienoic acid, methyl ester	5284421	−8.63	−8.06
13	Linolenic acid, methyl ester	5319706	−10.8	−8.86

## 4 Discussion

The present study investigated the antimicrobial potential and molecular docking interactions of *G. robusta* leaf extracts and explored their suitability for the green synthesis of copper nanoparticles (Cu‐NPs). The biosynthesis of Cu‐NPs was achieved using aqueous extracts of fresh and dried leaves of *G. robusta*. The reduction of Cu^2^^+^ ions was initially evidenced by a visible color change of the reaction mixture from brown to blackish‐green, indicating the formation of biogenic Cu‐NPs. Such color changes are commonly associated with the reduction of metal ions by plant‐derived phytochemicals. The phytochemical composition of the methanolic leaf extract was investigated using GC–MS analysis, which enabled the identification of volatile and semi‐volatile bioactive constituents that may contribute to antimicrobial activity and nanoparticle synthesis [30]. GC–MS analysis of the methanolic leaf extract identified several bioactive metabolites with retention times ranging from 9.623 to 29.376 min. The major constituents were 3‐O‐methyl‐D‐glucose (25.64%), palmitic acid (22.86%), and palmitic acid methyl ester (15.10%), followed by decane (9.40%), linolenic acid methyl ester (8.87%), and 1‐octadecyne (6.15%). Previous studies have reported antimicrobial, antioxidant, anti‐inflammatory, and membrane‐disrupting properties of fatty acids and their esters, particularly palmitic acid and linolenic acid derivatives [31, 32]. In addition, hydrocarbons such as decane and 1‐octadecyne contribute to the diverse phytochemical composition of the extract and may enhance its biological potential [33]. The abundance of fatty acids, esters, and carbohydrate derivatives suggests that *G. robusta* is a rich reservoir of biologically active metabolites. These compounds may collectively contribute to the antimicrobial activity of the extract through synergistic interactions and may also facilitate the reduction and stabilization of Cu‐NPs during green synthesis. Although the specific contribution of individual metabolites was not experimentally validated, the phytochemical profile provides valuable insight into the chemical basis of the observed bioactivity and supports the potential of *G. robusta* as a promising source of natural compounds for nanoparticle‐mediated antimicrobial applications [34]. The successful synthesis of Cu‐NPs was further confirmed by UV–Visible spectroscopy. Fresh leaf‐derived Cu‐NPs exhibited a characteristic surface plasmon resonance (SPR) peak at 550 nm, whereas dry leaf‐derived Cu‐NPs showed an SPR peak at 520 nm. These absorptions maxima fall within the characteristic range reported for plant‐mediated Cu‐NPs (500–600 nm), confirming nanoparticle formation [35]. The difference in SPR maxima between fresh and dry leaf‐derived Cu‐NPs may reflect variations in particle size, morphology, and surface chemistry resulting from differences in phytochemical composition after leaf drying.

FT‐IR spectroscopy was performed to identify the functional groups involved in the reduction and stabilization of Cu‐NPs synthesized using fresh and dry leaf extracts of *G. robusta*. The FTIR spectrum of fresh leaf‐derived Cu‐NPs exhibited prominent absorption bands at 1562.34 and 1056.99 cm^−^^1^. The band at 1562.34 cm^−^^1^ was attributed to aromatic C═C stretching and/or amide (−C═O−NH−) vibrations, indicating the presence of proteins, flavonoids, and polyphenolic compounds involved in nanoparticle stabilization [36]. The absorption band at 1056.99 cm^−^^1^ corresponded to C─O and C─N stretching vibrations associated with carbohydrates, glycosides, and other oxygen‐ and nitrogen‐containing biomolecules [37]. Similarly, the FTIR spectrum of dry leaf‐derived Cu‐NPs displayed absorption peaks at 1556.55 and 1060.85 cm^−^^1^, corresponding to comparable aromatic, amide, C─O, and C─N functional groups. The slight shifts in peak positions observed between fresh and dry leaf‐derived Cu‐NPs may be attributed to phytochemical changes induced during the drying process, which can influence the interaction of biomolecules with metal ions during nanoparticle formation [38]. Similar observations have been reported previously, where variations in phenolic and flavonoid contents affected nanoparticle size, stability, and surface properties [39]. Collectively, these findings suggest that phytochemicals present in *G. robusta* leaf extracts play important roles as both reducing and capping agents during Cu‐NP synthesis.

Scanning electron microscopy (SEM) further confirmed the successful synthesis of Cu‐NPs and provided insights into nanoparticle morphology and size distribution. SEM analysis revealed predominantly spherical nanoparticles for both fresh and dry leaf‐derived Cu‐NPs, consistent with previous reports on plant‐mediated synthesis of copper nanoparticles [40]. Fresh leaf‐derived Cu‐NPs exhibited particle sizes ranging from approximately 1–100 nm at the 1 µm scale and 20–50 nm at the 0.5 µm scale, with relatively uniform distribution and minimal aggregation. In contrast, dry leaf‐derived Cu‐NPs also displayed spherical morphology, but showed slight agglomeration in certain regions. These differences may be attributed to alterations in phytochemical composition during leaf drying, which can influence nanoparticle nucleation, growth, and stabilization processes [41]. The comparatively lower aggregation observed in fresh leaf‐derived Cu‐NPs indicates improved nanoparticle dispersion and stability. Energy‐dispersive x‐ray (EDX) analysis further confirmed copper as the predominant element in both nanoparticle preparations, demonstrating successful Cu‐NP formation. The presence of carbon and oxygen signals indicated the adsorption of plant‐derived organic compounds on the nanoparticle surface. These phytochemical residues likely contributed to nanoparticle stabilization and may also influence their biological activity. Similar observations have been reported for other plant‐mediated Cu‐NPs, where organic surface coatings enhance nanoparticle stability and functionality [42]. The crystalline nature of the synthesized Cu‐NPs was confirmed through x‐ray diffraction (XRD) analysis. The diffraction patterns obtained for both fresh and dry leaf‐derived Cu‐NPs exhibited characteristic peaks corresponding to the face‐centered cubic structure of copper, consistent with JCPDS file No. 04‐0836 [43]. The broad diffraction peaks indicated nanoscale crystallite dimensions, in agreement with the Scherrer equation, which predicts peak broadening with decreasing crystallite size [44]. No prominent peaks corresponding to CuO or Cu_2_O impurities were detected, indicating the high purity of the synthesized nanoparticles [45]. Minor variations in peak intensity and broadness between fresh and dry leaf‐derived Cu‐NPs may reflect differences in phytochemical composition affecting crystal growth and stabilization [46]. Collectively, the UV–Visible, FTIR, SEM, EDX, and XRD results provide complementary evidence for the successful green synthesis, stabilization, elemental composition, morphology, and crystallinity of Cu‐NPs mediated by *G. robusta* leaf extracts.

There are differences between the physicochemical properties of fresh leaf‐derived Cu‐NPs (FCu‐NPs) and dry leaf‐derived Cu‐NPs (DCu‐NPs), along with differences in biological activities. FCu‐NPs had lower aggregation, more uniform dispersion, and exhibited an SPR peak at 550 nm, while DCu‐NPs had an SPR peak at 520 nm and some degree of agglomeration. A slight difference in FTIR and XRD profiles also indicates that leaf drying impacted the phytochemicals used for synthesis and stabilization of nanoparticles. These differences were seen in the bioassays where the antibacterial activity of the FCu‐NPs was more effective while the activity of the DCu‐NPs were comparatively more effective against some fungal pathogens, such as *A. alternata*. The results presented here suggest that the chemical alterations that take place during leaf‐drying have the potential to affect the properties of the nanoparticles and their biological activity.

The antimicrobial activities of the methanolic crude extract (MGR), fresh leaf‐derived Cu‐NPs (FCu‐NPs), and dry leaf‐derived Cu‐NPs (DCu‐NPs) were evaluated against selected bacterial and fungal pathogens. The synthesized Cu‐NPs exhibited enhanced antimicrobial activity compared with the crude extract, indicating that nanoparticle formation improved biological efficacy. This enhanced activity may be attributed to the increased surface area, improved cellular interactions, and higher reactivity of Cu‐NPs [47]. Previous studies have suggested that Cu‐NPs exert antimicrobial effects through multiple mechanisms, including membrane disruption, reactive oxygen species (ROS) generation, enzyme inhibition, and interactions with intracellular biomolecules [48]. However, these mechanisms were not directly investigated in the present study and therefore remain potential explanations based on existing literature. The observed differences between FCu‐NPs and DCu‐NPs may be associated with variations in particle morphology, aggregation state, and phytochemical capping resulting from the use of fresh and dried leaf extracts. Fresh leaf‐derived Cu‐NPs generally demonstrated stronger antibacterial activity against several tested bacterial strains, which may reflect differences in nanoparticle characteristics and surface‐associated phytochemicals. In contrast, the crude methanolic extract exerted antimicrobial effects through the combined action of its phytochemical constituents, including phenolics, flavonoids, and tannins, which have previously been reported to interfere with microbial metabolism, cell wall integrity, and oxidative balance [49].

The antifungal assay revealed notable differences among treatments. The methanolic extract exhibited strong activity against *F. oxysporum*, producing a maximum inhibition zone of 40 ± 1.63 mm at 12 µg/mL, while *M. phaseolina* displayed the lowest EC_50_ value (2.8506 µg/mL), indicating high susceptibility. Fresh leaf‐derived Cu‐NPs demonstrated considerable antifungal activity, with inhibition zones ranging from 12 ± 0.81 to 36 ± 1.63 mm. Notably, FCu‐NPs showed enhanced activity against *F. solani*, as reflected by the lower EC_50_ value (6.0683 µg/mL) compared with the crude extract (9.7543 µg/mL). Dry leaf‐derived Cu‐NPs also exhibited substantial antifungal activity, with inhibition zones ranging from 25 ± 0.81 to 30.33 ± 1.24 mm. Among the tested pathogens, DCu‐NPs displayed comparatively stronger activity against *A. alternata*. These differences suggest that the phytochemical composition of fresh and dry leaf extracts may influence nanoparticle characteristics and, consequently, antifungal performance [50, 51]. While direct comparisons among studies should be interpreted cautiously due to differences in nanoparticle synthesis protocols, physicochemical characteristics, microbial strains, and assay conditions, the present findings indicate that *G. robusta*‐derived Cu‐NPs exhibit antimicrobial activities comparable to those reported for other green‐synthesized Cu‐NPs and demonstrate promising efficacy under the experimental conditions employed in this study.

The antibiofilm activity of *G. robusta*‐derived Cu‐NPs was assessed through anti‐pellicle assays against *E. coli*, *Acinetobacter* sp., and *Citrobacter* sp. Biofilm formation is a major virulence factor that enhances microbial resistance to antimicrobial agents [52]. The crude methanolic extract exhibited moderate antibiofilm activity, particularly at higher concentrations, which may be attributed to phenolic and flavonoid compounds that interfere with quorum sensing and bacterial adhesion processes [53]. In contrast, both FCu‐NPs and DCu‐NPs demonstrated stronger antibiofilm activity, with substantial pellicle disruption observed at higher concentrations. FCu‐NPs showed pronounced activity against *E. coli*, whereas DCu‐NPs exhibited stronger inhibition of *Acinetobacter* sp. and *Citrobacter* sp. These findings indicate that nanoparticle formulation enhanced antibiofilm efficacy relative to the crude extract. The superior antibiofilm activity of Cu‐NPs may be associated with their nanoscale size, large surface area, and enhanced interaction with microbial cells. Previous studies have suggested that Cu‐NPs can interfere with the initial stages of biofilm development by reducing microbial adhesion to surfaces through alterations in cell membrane integrity and surface charge characteristics [54]. In addition, Cu‐NPs have been reported to generate reactive oxygen species (ROS), leading to oxidative stress that can impair cellular functions involved in adhesion, extracellular polymeric substance (EPS) production, and biofilm maturation. Since EPS forms the structural matrix that protects biofilm‐associated cells, disruption of its synthesis may weaken biofilm architecture and increase microbial susceptibility to antimicrobial agents. Cu‐NPs may also interfere with quorum sensing pathways, which regulate microbial communication, surface colonization, and coordinated biofilm formation. Furthermore, the small size of Cu‐NPs may facilitate penetration into the biofilm matrix, allowing direct interaction with embedded cells and contributing to pellicle disruption and reduced biofilm stability [55]. The observed strain‐specific responses may reflect differences in biofilm architecture, EPS composition, cell surface properties, and intrinsic resistance mechanisms among bacterial species [56, 57]. For example, variations in membrane structure and biofilm density may influence nanoparticle penetration and susceptibility to oxidative damage. However, the exact molecular mechanisms responsible for the antibiofilm and antipellicle activities observed in the present study were not directly investigated and therefore require further experimental validation through gene expression, microscopy, and biofilm matrix analyses.

Molecular docking was conducted to provide mechanistic insight into the antimicrobial activity of *G. robusta*‐mediated Cu‐NPs. The selected targets, penicillin‐binding protein (1EA1) and sterol 14α‐demethylase (3VSL), are essential enzymes involved in bacterial cell wall synthesis and fungal ergosterol biosynthesis, respectively. Inhibition of these proteins may impair microbial growth and survival. Among the tested phytochemicals, linolenic acid methyl ester exhibited the strongest binding affinity against 1EA1 (−10.80 kcal/mol), whereas palmitic acid showed the highest affinity toward 3VSL (−11.27 kcal/mol). Other compounds, including 3,7,11,15‐tetramethyl‐2‐hexadecen‐1‐ol and 3‐O‐methyl‐D‐glucose, also demonstrated favorable interactions with the selected targets. Comparison with the reference inhibitors revealed that although the phytochemicals generally displayed lower binding affinities than HEM (−19.58 kcal/mol) and CEF (−14.09 kcal/mol), several compounds exhibited docking scores comparable to TPF (−10.05 kcal/mol), indicating promising inhibitory potential. The interaction profiles observed in Figures S29 and S30 suggest that these compounds bind within the active sites of the target proteins through hydrophobic contacts and hydrogen‐bonding interactions. These findings support the in vitro antimicrobial activity and indicate that fatty acids, fatty acid esters, and terpenoid derivatives identified in *G. robusta* may contribute to microbial inhibition. Nevertheless, molecular docking is a predictive computational approach, and further enzymatic, molecular dynamics, and in vivo studies are required to validate the proposed mechanisms of action.

## 5 Conclusion

In this study, copper nanoparticles (CuNPs) were successfully synthesized and characterized using fresh and dried leaf extract of *G. robusta*, which was done in a green, eco‐friendly, and sustainable manner. The novelty of this work is the use of *G. robusta* leaf extract as a natural reducing and stabilizing agent for the preparation of Cu‐NPs, which are environmentally friendly instead of the conventional chemical synthesis. A detailed characterization by UV–Visible spectroscopy, FT‐IR, SEM, EDX, and XRD confirmed the successful formation of stable, crystalline, and well‐dispersed CuNPs. Under in vitro conditions used in this study, the synthesized nanoparticles showed significant antimicrobial activities, including antibacterial, antifungal, antipellicle, and antibiofilm activities, as indicated by low EC_50_ values, which indicated strong antimicrobial activity against the tested clinically and agriculturally relevant pathogens. In addition, molecular docking studies indicated the potential interactions of selected bioactive compounds with microbial target proteins, offering preliminary insights into the potential mode of action of the antimicrobial effects. In summary, the results suggest that *G. robusta* mediated CuNPs are potential candidates for further antimicrobial studies and the importance of this plant as a sustainable source for the production of green nanotechnology products. Still, further studies, such as cytotoxicity testing, biocompatibility testing, mechanistic studies, formulation development, and in vivo validation, need to be conducted before any practical biomedical or agricultural applications can be established.

## Author Contributions

Conceptualization and resources: **Gul Naz**. Data curation: **Barkat Ullah** and **Atta Ur Rahman**. Writing – original draft, Formal analysis, and Visualization: Atta Ur Rahman. Formal analysis and Investigation: **Arshad Iqbal**. Methodology: Gul Naz, Barkat Ullah, and Atta Ur Rahman. Validation: Arshad Iqbal. Writing – review and editing: **Atta Ur Rahman** and **Samra Irem**. Data Curation: **Arshad Iqbal** and **Wajih Ud Din**. Software: **Atta Ur Rahman**, **Barkat Ullah**, and **Dure‐E‐Najaf Khan**. All authors contributed significantly, and have read and agreed to the published version of the manuscript.

## Ethical Statement

The present research does not meet the ethical approval for the use of data. There was no test organisms involved in any of the sections of the present research.

## Conflicts of Interest

The authors declare no conflicts of interest.

## Supporting information




**Supporting File 1**: cbdv71632‐sup‐0001‐SuppMat.docx.

## Data Availability

The data, such as the source file associated with this finding, are available from the corresponding author upon request.
