# Supplementary material for: Green Synthesis of Copper Nanoparticles Using Aqueous Extract of Grevillea robusta: In Vitro Antimicrobial and In Silico Approach
Source: Chem Biodivers. 2026 Aug 26;23(8):e71632. doi: 10.1002/cbdv.71632 (PMC13510125; doi:10.1002/cbdv.71632)
Supplement: Supplementary file 1 [file CBDV-23-e71632-s001.docx]

**Figure S1:** Images showing fresh leaves of *G. robusta* used in the study

**Figure S2:** Aqueous Extract of leaf of *G. robusta*

**Figure S3: Preparation of Copper Sulfate Solution**

**Figure S4: GC-MS Chromatogram of plant crude extract**

**Figures S5–7.** Antipellicle activity of ciprofloxacin (positive control), crude extract of Grevillea robusta, and fresh and dry leaf-mediated copper nanoparticles (Cu-NPs) against Escherichia coli, Acinetobacter sp., and Citrobacter sp. at concentrations of 3, 6, 9, and 12 µg/mL.

**Figures S8-9**. Antibiofilm activity of the crude extract and fresh and dry leaf-mediated copper nanoparticles (Cu-NPs) synthesized from Grevillea robusta: (Figure 17) biofilm inhibition after 24 h incubation and (Figure 18) biofilm formation following staining.
